# Comparative sperm recovery rate after density gradient centrifugation with two media for *in vitro* fertilization

**DOI:** 10.5935/1518-0557.20220008

**Published:** 2023

**Authors:** Nilma De Souza Fernandes, Carla Grasiele Da Silva, Gean Pier Panizzon, Paula Motta Almodin Cerialle, Vânia Cibele Minguetti Câmara, Moacir Rafael Radaelli, Carlos Gilberto Almodin

**Affiliations:** 1 Materbaby - Reprodução Humana e Genética. Maringá, Brazil; 2 Ingá Materiais Médico Hospitalares LTDA, Maringá, Brazil; 3 Departamento de Análise Farmacêutica, Universidade Estadual de Maringá - UEM, Maringá, Brazil; 4 Departamento de Urologia, Escola de Medicina, Faculdade Ingá, Maringá, Brazil

**Keywords:** Density gradient, spermatozoa, fertilization

## Abstract

**Objective:**

To compare the efficacy of two density gradient centrifugation media for retrieving spermatozoa from semen samples by evaluating the total motile sperm count (TMSC) and the percentage recovery.

**Methods:**

Twenty-two men with different sperm counts participated in the study. The samples were divided into two equal aliquots and processed using the commercial ISolate Sperm Separation Medium (Irvine Scientific, United States) and the GV Gradiente (IngáMed, Brazil). After separation, samples were counted and evaluated for motile sperm recovery.

**Results:**

The mean TMSC in the fresh sample was 19.65±21.08 million/mL. After the ISolate separation the TMSC was 6.71±7.29 million/mL, and for the GV Gradiente it was 6.27±6.82 million/mL. The percentage of motile spermatozoa recovered was 36.47%±21.61 for ISolate and 35.22%±21.24 for the GV Gradiente (*p*>0.05). The samples from 6 oligospermic patients (27%) were evaluated separately and the TMSC for ISolate was 4.83±2.92 million/mL, and for the GV Gradiente, it was 4.16±3.12 million/mL (*p*=0.54). When evaluating only normospermic patient samples, the TMSC for ISolate was 9.05±7.29 million/mL, and for the GV Gradiente, it was 8.47±6.79 million/mL (*p*=0.83).

**Conclusions:**

There was no statistical difference in retrieving motile sperm using the GV Gradiente and the ISolate Separation Medium.

## Introduction

To perform assisted reproduction techniques (ART), sperm must first be processed by selecting for morphologically normal spermatozoa with high motility in order to maximize the concentration of good quality sperm. The preparation also removes cell debris, including epithelial cells and leukocytes (Malvezzi *et al*., 2014; Ahammad *et al*., 2018). The semen preparation procedure is therefore an important factor that directly affects the outcome of infertility treatment.

Several protocols, based on sperm migration, have been used for sperm enrichment. These include techniques such as the ‘swim up’ technique in which highly motile sperm migrate from the seminal fluid into culture medium (Beydola *et al*., 2013). Although relatively easy and accessible, the techniques based on sperm migration have been questioned regarding the sperm recovery for patients with low sperm counts and seminal motility (Malvezzi *et al*., 2014). An alternative for cases of patients with oligospermia is the preparation of sperm by density gradient centrifugation (DGC) using media with a low osmolarity. This approach leads to the separation of sperm cells based on their density (Bras *et al*., 1996).

Morphologically normal sperm have a higher density than immature or morphologically abnormal sperm. Semen is placed on top of media layered at different dilutions in a disposable centrifuge tube and, after centrifugation, each sperm cell will be located at the level of the gradient that corresponds to its density. As a result, highly motile, morphologically normal, and viable sperm form a pellet at the bottom of the tube, leukocytes and cells debris will be trapped at the interface between the seminal plasma and the more dilute upper phase, and morphologically abnormal sperm with low motility will be at the interface between the layers (Ahammad *et al.*, 2018). This method is considered simple, reproducible, and efficient for isolating high quality sperm for use in ART and has been widely used in cases of normospermic patients (Ahammad *et al.*, 2018; Jayaraman *et al.*, 2012; Yang *et al.*, 2015).

Different DGC media have been proposed to improve the efficiency of the method. Initially, Percoll was widely used, which is composed of silica particles coated with polyvinylpyrrolidone (PVP). However, due to the toxicity of PVP, its use in humans has been discontinued (Mousset-Siméon *et al*., 2004; Ahammad *et al*., 2018). Since then, iodixanol or colloidal solutions of silica particles coated with silane have been tested as potential alternatives (Mousset-Siméon *et a*l., 2004; Lee & Jee, 2019).

Our group proposes another density gradient sperm separation medium, GV Gradiente produced by IngáMed, Brazil. GV Gradiente is a buffered balanced saline solution composed of a colloidal suspension of silica particles stabilized with hydrophilic silane. Thus, the aim of this study was to evaluate the sperm recovery of GV Gradiente by comparing with the commercial medium, ISolate Sperm Separation Medium (Irvine Scientific, USA). The total concentration of sperm and motile forms recovered were evaluated following semen processing by these DGC methods.

## Materials and Methods

This study was approved by the Research Ethics Committee of UNINGA University (Protocol: 4.728.627). Semen samples were obtained from 22 men aged 29 to 47 years, through masturbation into a wide and sterile bottle (IngáMed, Brazil) after a 2 to 7-day period of abstinence. The samples remained at room temperature for 40-60 minutes for liquefaction. After complete liquefaction, the samples were counted and then divided into two equal parts for sperm separation by the DGC method using 1) the ISolate Sperm Separation Medium (Irvine Scientific, USA) and 2) the GV Gradiente (IngáMed, Brazil).

The semen was deposited on a 50/90% ISolate tube and on a 45/90% GV Gradiente tube, and then centrifuged at 300 × g for 20 minutes. The supernatant was discarded, and the pellet consisting of sperm was washed with GV HEPES (IngáMed, Brazil) at 300 × g for 10 minutes. After washing, the supernatant was again discarded, and the pellet containing the isolated sperm was resuspended in 0.5 mL of GV HEPES (IngáMed, Brazil). After the sperm separation methods were applied, the sperm motility parameter in both samples(ISolate and GV Gradiente) was evaluated by the manual counting method in a Makler counting chamber under phase contrast microscopy with 400x total magnification.

Data are presented as mean ± standard deviation. The Kruskal-Wallis test followed by Dunn’s multiple comparisons test were used to compare the semen parameters of the fresh samples and after each DGC medium used. The Mann-Whitney test was used to compare the results from the two different DGC media. The Z test compared the recovery proportions between the DGC media. The frequency distribution of samples was used to confirm sample equivalence. In all tests, *p*<0.05 was considered statistically significant. GraphPad Prism 8.0.1 software was used for the analysis and generating the graphs.

## Results

### Characteristics of the sample population

The men participating in the study had a mean age of 37.36 ± 4.35 years (95% CI 35.43-39.30). The fresh total sperm count averaged 36.99 ± 30.95 million/mL (95% CI 23.26-50.71). Total sperm count did not correlate with the age of the participants (r=0.20). The total motile sperm count (TMSC) averaged 19.65 ± 21.08 million/mL (95% CI 10.30-29.99) and had no correlation with age (r=0.14). The mean percentage of motile sperm in relation to the total was 55.18 ± 27.43% (95% CI 43.02-67.34).

**Figure 1:**
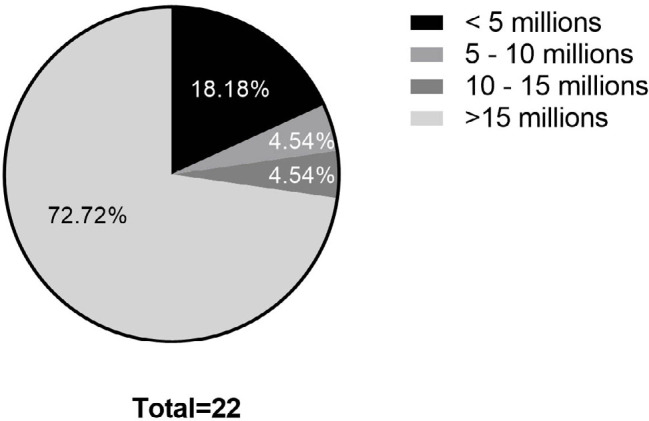
Characteristics of the sample population based on the total number of spermatozoa. The total sperm count was used to calculate the percentage of men who had oligospermia and normospermia in the population evaluated.

Of the 22 men, six (27.8%) were characterized with oligospermia (<15 million sperm per mL of semen) according to the World Health Organization (WHO,2010). Among these, four (18.8%) had severe oligospermia (below 5 million/mL), one (4.5%) had moderate oligospermia (between 5 and 10 million), and one (4.5%) had medium oligospermia (between 10 and 15 million). The majority, 16 patients (72.7%), were classified as having normospermia (>15 million) (Figure 1).

### Comparison between the total motile sperm count (TMSC) before and after separation with ISolate and GV Gradiente media

**Figure 2:**
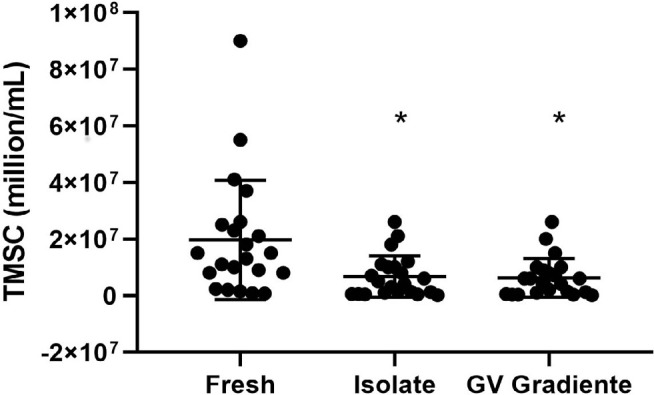
Total count of motile sperm before and after separation with the gradient media, ISolate and GV Gradiente. TMSC, total motile sperm count; * indicates statistical difference compared to fresh semen, *p*<0.001.

The values of motile sperm in the fresh sample and after processing by the two DGC media were compared and evaluated (Figure 2). The mean TMSC in the fresh sample was 19.65 ± 21.08 million/mL (95% CI 10.3-28.9). For the ISolate medium, the mean TMSC was 6.71 ± 7.29 million/mL (95% CI 3.4-9.9) and for the GV Gradiente it was 6.27 ±6.82 million/mL (95% CI 3.2-9.3).

### Sperm recovery after separation with ISolate and GV Gradiente media

**Figure 3:**
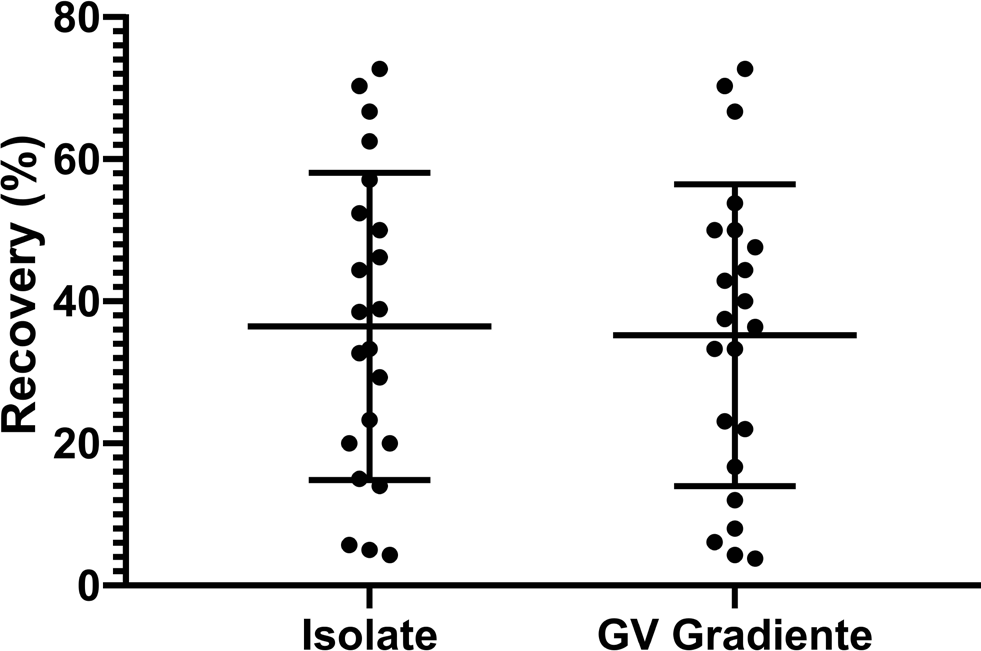
Percentage of motile spermatozoa after separation with the density gradient media, ISolate and GV Gradiente, *p*=0.93.

The mean rate of motile sperm retrieved was 36.47% ± 21.61 (95% CI 26.89-46.05) for ISolate and 35.22 % ± 21.24 (95% CI 25.80-44.64) for GV Gradiente (Figure 3). The comparison between the two groups is shown in Figure 3, where *p*=0.93, although there was no significant difference.

Another evaluation to verify the separation efficiency by the DGC media was the analysis of samples considered oligospermic and normospermic separately. Sperm from six patients (27%) with sperm counts below 15 million per mL were compared after separation with ISolate and GV Gradiente. The mean TMSC for ISolate was 4.83 ± 2.92 million/mL (95% CI 1.76-7.90) and for the GV Gradiente, the value was 4.16 ± 3.12 million/mL (95% CI 1.32-19.18). When evaluating only patients with normospermia, the mean for ISolate was 9.05 ± 7.29 million/mL (95% CI 0.51-12.94)and for the GV Gradiente, the value was 8.47 ± 6.79 million/mL (95% CI 0.48-12.09).

**Figure 4:**
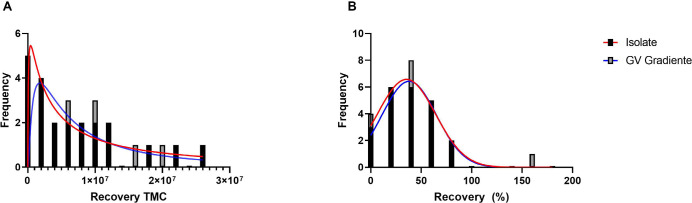
Frequency histograms of the distribution of the evaluated samples. (A) Frequency histogram of motile sperm counts (TMSC) after separation with density gradient media evaluated with a log-normal distribution curve. (B) Frequency histogram of the percentage of sperm recovered after separation with density gradient media with a normal distribution curve.

The distribution of samples after separation was evaluated in order to confirm the equivalence of the DGC media in relation to the retrieval of motile sperm. The frequency histograms show the TMSC distribution after separation with the ISolate and GV Gradiente media (Figure 4A), as well as the recovery percentage (Figure 4B). Data had normal distribution. The similarity of the values obtained in the recovery rate after separation with the ISolate and GV Gradiente media is observed by the Gaussian curve plotted in the graph.

## Discussion

One of the main challenges of reproductive medicine is to ensure that the highest quality sperm are used in ARTs. The aim of this study was to determine if there was a difference in sperm recovery after processing with two different DGC media: ISolate and GV Gradiente.

Sperm preparation techniques must be operationally simple and cost-effective in order to suit the routine procedures of human reproduction clinics. It should result in a sample that is enriched with high quality sperm in the shortest possible time. In addition to removing low-quality sperm, including immotile sperm, sperm preparation techniques must allow the elimination of other cells, such as leukocytes and bacteria, as well as toxic or bioactive substances like reactive oxygen species (ROS) (Malvezzi *et al*., 2014; Boomsma *et al.*, 2019).

The use of density gradients allows the isolation of a high percentage of morphologically normal and motile human sperm from seminal plasma, and these are also free of debris, dead sperm, and non-sperm cells. Method variants consist of different gradient types, for example continuous or discontinuous, as well as different substances used to generate the density gradient: silica particles coated with PVP or Percoll, or commercial variants such as IxaPrep^®^ (MediCult, Copenhagen, Denmark), Sil-Select^®^ (FertiPro NV, Beernem, Belgium), PureSperm^®^ (NidaCon Laboratories AB, Gothenburg, Sweden), or ISolate^®^ (Irvine Scientific, Santa Ana, CA, USA). Commercial media are currently used in human clinics to replace Percoll due to its toxicity and side effects on sperm function (Mousset-Siméon *et al*., 2004; Ahammad *et al*., 2018; Henkel & Schill, 2003).

Both sperm separation media used in this study consist of a colloidal suspension of silica particles stabilized with hydrophilic silane buffered with HEPES. They were purchased ready-to-use. In addition to having the same density agent and buffering system, the qualitative composition reported by the manufacturers is composed of sodium chloride, potassium chloride, magnesium sulfate, potassium phosphate, calcium chloride, glucose, sodium pyruvate, sodium lactate, and sodium bicarbonate. In this way, it is also possible to verify their similarity in relation to the composition of salts, ions, and energetic substrates.

The results demonstrated that sperm separation using the two DGC media did not show any significant difference in terms of the recovered motile sperm concentration (*p*=0.80). The percentage of recovery did not differ either (*p*=0.93). Furthermore, the recovery percentage for ISolate was similar to that demonstrated by Malvezzi *et al*. (2014) (48.9±18.7%).

The current study is significant because it assessed sperm quality after two different sperm preparation media used under identical conditions. Furthermore, samples from patients with oligospermia and normosperia were assessed separately under these conditions. It is believed that the effects of gradient media may be more expressive in samples with a smaller amount of sperm or semen of lower quality (Lee & Jee, 2019). Here it was possible to demonstrate that the sperm retrieved after separation of oligospermic patient samples on the GV Gradiente did not differ compared to that following the ISolate separation (*p*=0.54). When only normospermic samples were evaluated, there was also no statistical difference between the ISolate and the GV Gradiente media (*p*=0.83). These results demonstrate that the media have the same efficiency for preparing sperm from low concentration samples, as well as normal count or heterogeneous samples.

Several other methodologies have been developed with the aim of improving the quality of isolated sperm for use in IVF, such as selection methods depending on sperm motility using microfluidic systems that are based on physical sperm properties such as chemotaxis, rheotaxis, and thermotaxis. Although promising, these systems have not yet been shown to be useful for their routine application in clinical practice and seem to be effective only in some very specific cases of male infertility (Oseguera-López *et al*., 2019). Thus, the density gradient method is still the technique of choice for sperm selection in most assisted reproduction centers. As a technique widely used by assisted reproduction centers, the use of nationally sourced inputs is essential. GV Gradiente is a product manufactured in Brazil and easy to purchase, and as it is a national product, it is not subject to the risk of physical-chemical alterations due to long transportation times.

## Conclusion

The results shown here, in relation to ISolate, GV Gradiente has comparable efficacy in sperm separation, suggesting that this product would be ideal for performing the DGC technique in the country.
